# Effects of Combined Caffeine and *Rhodiola rosea* Supplementation on Repeated Aerial Duel Performance and Neck Neuromuscular Function in Soccer Players

**DOI:** 10.3390/nu18091339

**Published:** 2026-04-23

**Authors:** Yue Dou, Ziyi Feng, Hengquan Xu, Hexin Ma, Yuewei Jiang, Xinping Lyu, Bolin Han, Shuning Liu, Chang Liu, Dingmeng Ren

**Affiliations:** 1 China Football College, Beijing Sport University, Beijing 100084, China; 2023210540@bsu.edu.cn (Y.D.);; 2 School of Physical Education, Xi’an University, Xi’an 710065, China; 3 School of Sport and Health Science, Xi’an Physical Education University, Xi’an 710068, China; 4 School of Education, Communication & Society, King’s College London, London WC2R 2LS, UK; 5 China Institute of Sport Science, General Administration of Sport of China, Beijing 100061, China; 6 Faculty of Engineering, The Hong Kong Polytechnic University, Hong Kong 999077, China; 7 School of Sport Science, Beijing Sport University, Beijing 100084, China

**Keywords:** ergogenic aids, heading performance, neuromuscular control, repeated-effort performance, fatigue regulation

## Abstract

Background: Soccer aerial duels require rapid take-off, repeated-performance maintenance, and effective head–neck control under physically demanding conditions. This study examined the effects of caffeine (CAF), Rhodiola rosea (RHO), and their combination on repeated aerial duel performance and neck neuromuscular function in male collegiate soccer players. Methods: Ninety-six players were randomly assigned, in a double-blind, placebo-controlled, parallel design, to placebo control (CTR), RHO, CAF, or RHO + CAF groups (*n* = 24 each) for 4 weeks. CAF was acutely administered at 3 mg·kg^−1^ before testing, whereas RHO was chronically supplemented at 2.4 g·day^−1^. Outcome measures included countermovement jump height, early take-off impulse, repeated heading contact height, ball exit velocity, heading duel success rate, neck maximal voluntary isometric contraction, and session rating of perceived exertion (session-RPE). Results: Significant group × time or group × repetition effects were observed for CMJ height (*p* = 0.0034), early take-off impulse (*p* = 0.0007), and post-intervention repeated heading contact height (*p* < 0.0001), with additional significant effects across heading-specific, neck strength, duel-success, and perceived-load outcomes. CAF was mainly associated with improved take-off-related explosive performance and duel success, whereas RHO was mainly associated with lower perceived exertion and better maintenance of heading contact height during the later repeated trials. Combined RHO + CAF supplementation produced the broadest pattern of benefits across explosive output, ball-contact performance, duel success, and multidirectional neck strength. Conclusions: These findings suggest that, in male collegiate soccer players, CAF and RHO may contribute differently to repeated aerial duel-related performance, and their combination may offer broader sport-specific benefits under repeated high-intensity demands.

## 1. Introduction

In high-intensity intermittent sports such as soccer, match play is prolonged (90 min) and characterized by frequent transitions between attack and defense, requiring players to repeatedly perform key actions such as sprinting, jumping, changing direction, and engaging in high-intensity physical duels throughout the game. Heading and aerial duels are common in set-piece contests, long-ball clearances, and second-ball situations following aerial play [1]. Their competitive value depends on more than simply “jumping the highest” on a single occasion. Players must also be able to repeatedly generate rapid take-off performance, maintain body control, and convert these actions into effective ball-contact outcomes and duel success [2]. Aerial duel success may therefore be understood as a performance chain. Rapid lower-limb force production during take-off initiation may provide an early advantage in reaching the ball, whereas efficient momentum transfer and stable head-neck-torso control at ball contact are likely to influence ball exit velocity and the final duel outcome [3]. In this context, the stabilizing demands on the head and neck extend beyond sagittal-plane flexion-extension control and also include frontal-plane stability. During mid-air collision, asymmetric perturbation, and off-center ball contact, lateral neck control may help maintain head posture, resist perturbation, and preserve directional accuracy of ball contact [4].

Because aerial duel performance depends on both rapid initiation and the ability to maintain execution quality across repeated efforts, it provides a relevant context for considering nutritional strategies that may support these different task demands [5]. In this regard, caffeine (CAF) and Rhodiola rosea (RHO) are two supplementation strategies that have received substantial attention in sports nutrition, although their proposed functional roles appear to differ [6]. As a classic adenosine receptor antagonist, CAF is thought to enhance rapid force production primarily by attenuating central inhibition, increasing neural drive, and improving the recruitment efficiency of high-threshold motor units during movement initiation [7]. In contrast, RHO, a plant-derived adaptogen, contains bioactive compounds believed to possess antioxidant, anti-fatigue, and stress-regulating properties and may therefore be more likely to attenuate fatigue accumulation during repeated high-intensity loading and help maintain performance during exercise. Accordingly, in a soccer-specific task such as aerial dueling, which requires both explosiveness and repeatability, CAF and RHO may not act on the same functional components but instead may show distinct application potential in rapid initiation and fatigue buffering, respectively [8].

However, the current evidence remains insufficient to clearly explain the specific functional pathways through which nutritional interventions may influence aerial duel performance in soccer [9]. Although the ergogenic effects of CAF on rapid force production and neuromuscular excitation have been widely supported, its benefits for sport-specific performance under sub-fatigued or fatigued conditions remain inconsistent, with considerable inter-individual variability [10]. By comparison, existing studies on RHO have focused mainly on endurance capacity, anti-fatigue effects, or post-exercise recovery. In contrast, direct evidence regarding its role in maintaining repeated performance during high-intensity, intermittent, and confrontational tasks remains limited [11]. More importantly, previous research has not adequately tested a practically meaningful possibility. One possibility is that CAF may preferentially enhance rapid mobilization and explosive output during the initiation phase of aerial actions, whereas RHO may preferentially attenuate fatigue accumulation and preserve performance stability across subsequent repeated efforts. Whether combined supplementation with CAF and RHO can provide complementary benefits across both the “initiation” and “maintenance” phases has not yet been directly established.

At the same time, research specifically targeting soccer-related aerial duel tasks remains limited. Existing literature has rarely established an integrated evaluation chain spanning early take-off kinetics, sport-specific heading performance, and final duel outcomes, making it difficult to determine whether a given intervention primarily improves peak explosiveness in a single action or enhances performance stability across repeated executions [12]. Particularly in heading and aerial contest situations, performance depends not only on lower-limb explosive capacity but also on the stabilizing role of the head-neck-torso chain during aerial contact, body collision, and ball-control processes [13]. Nevertheless, previous studies have focused predominantly on general explosive performance or single-direction strength measures, with insufficient attention given to multi-directional neck maximal voluntary isometric contraction (MVIC), which is closely related to aerial movement control. Consequently, the current field lacks both direct tests of the potential complementary mechanisms of CAF and RHO and a systematic assessment framework capable of integrating lower-limb kinetics, sport-specific heading performance, and multi-directional neuromuscular characteristics of the neck [14].

Therefore, the present study aimed to compare the effects of CAF, RHO, and their combined supplementation on soccer-specific heading and aerial duel performance in male collegiate soccer players, with particular emphasis on repeated-performance maintenance and neck neuromuscular function. In addition, multi-directional neck maximal voluntary isometric contraction was incorporated into the assessment framework to reflect neuromuscular characteristics associated with head-neck stability during aerial actions. We hypothesized that: (1) CAF supplementation alone would primarily improve early kinetic performance during take-off initiation; (2) RHO supplementation alone would mainly exert its effects by reducing perceived exertion and supporting execution stability across repeated efforts; and (3) combined supplementation (RHO + CAF) would produce broader and more integrated improvements across multiple sport-specific components, resulting in superior overall effects on explosive output, head-neck stability-related strength, and the translation of these qualities into sport-specific performance outcomes.

## 2. Materials and Methods

### 2.1. Participants

A total of 96 male soccer players from the varsity teams of Beijing Sport University were recruited for this study. Baseline participant characteristics are presented in Table 1. All participants completed the Physical Activity Readiness Questionnaire (PAR-Q), and the screening results indicated that they were suitable for high-intensity physical testing and training.

The inclusion criteria were as follows: (1) engagement in systematic soccer-specific training for at least 6 years, with stable recent training exposure (training frequency ≥ 3 sessions per week); and (2) good physical condition, enabling completion of the high-intensity exercise testing and training procedures required in the present study, as confirmed by successful PAR-Q screening. According to the Participant Classification Framework proposed by McKay et al., the present cohort was retrospectively classified as Tier 3 (highly trained/national level) based on their long-term systematic soccer training history, regular structured training exposure, and participation in varsity-level competitive soccer [15].

To minimize external confounding factors and ensure participant safety, the following exclusion criteria were applied: (1) a previous or current history of neuromuscular disorders or severe cardiovascular or musculoskeletal disease; (2) current use of medications that could potentially interact with *Rhodiola rosea* or caffeine (e.g., antidepressants or central nervous system stimulants), or the presence of clear contraindications to such supplementation; (3) regular consumption of caffeine-containing products (including coffee and tea), other ergogenic aids, or alcohol-containing products within the 3 months preceding the start of the study; and (4) smoking. All of the above information was confirmed by the research team during enrollment through questionnaires and interviews.

Before the experiment began, the research team provided all participants with a detailed explanation of the study purpose, experimental procedures, and potential risks, and written informed consent was obtained from all participants. During the intervention period, participants were instructed to maintain their habitual diet and training routines and to strictly comply with the research team’s requirements regarding supplement intake and the restriction of prohibited substances. No serious adverse events related to nutritional supplementation were reported during the study. No significant between-group differences were found at baseline for age, height, body mass, or training experience (all *p* > 0.05).

### 2.2. Ethics Approval and Trial Registration

The study protocol was reviewed and approved by the Ethics Committee of Beijing Sport University (approval No. 2026038H). All study procedures were conducted in accordance with the ethical principles for human research outlined in the Declaration of Helsinki. Before formal participation, all participants provided written informed consent and were clearly informed that they could withdraw from the study at any stage without any adverse consequences. To enhance the methodological rigor of study implementation and outcome reporting, the trial was designed and conducted with reference to the Consolidated Standards of Reporting Trials (CONSORT) statement and was registered at ClinicalTrials.gov (Identifier: NCT07458594; date of registration 4 March 2026).

### 2.3. Study Design

This study employed a randomized, double-blind, placebo-controlled, parallel-group design to systematically evaluate the effects of caffeine (CAF) and *Rhodiola rosea* (RHO), administered alone or in combination, on soccer-specific aerial duel performance.

The total intervention period was 4 weeks. After completion of the baseline assessment (Pre), participants were randomly allocated, using an allocation concealment procedure, by an independent researcher who was not involved in participant recruitment, data collection, outcome assessment, or statistical analysis, into one of four parallel groups: placebo control group (CTR), *Rhodiola rosea* group (RHO), caffeine group (CAF), and combined *Rhodiola rosea* plus caffeine group (RHO + CAF), with 24 participants in each group. Baseline characteristics were also checked for group comparability before intervention, and no significant between-group differences were observed. Throughout the study, participants, test administrators, and outcome assessors were all blinded to group allocation.

**Figure 1:**
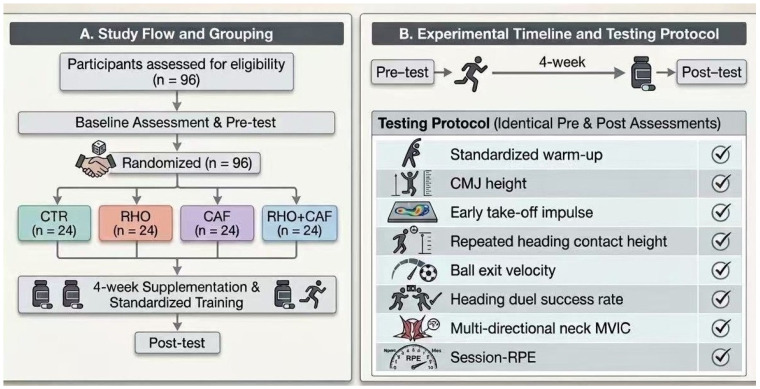
Diagram of the Experimental Procedure.

All participants completed the post-intervention assessment (Post) after the 4-week intervention period. To ensure comparability between Pre and Post measurements, the testing time window, test order, equipment setup, operational procedures, and recovery intervals at Post were kept identical to those used at Pre. The overall experimental procedure is illustrated in Figure 1.

### 2.4. Supplement Preparation and Supplementation Protocol

A randomized, double-blind, placebo-controlled supplementation strategy was used in this study [16]. Caffeine (CAF) capsules were purchased from Nutricost (Vineyard, UT, USA), and *Rhodiola rosea* (RHO) capsules were obtained from Tongrentang Pharmaceutical Co., Ltd. (Beijing, China). Placebo capsules contained no active ingredients and were primarily filled with starch; their appearance and packaging were matched to those of the active supplements. To ensure blinding, all supplements were uniformly repackaged, coded, and distributed by an independent researcher who was not involved in subsequent data collection or statistical analysis, such that capsules under different conditions were identical in appearance, size, color, texture, and packaging. To minimize potential batch-related variation, all supplements used during the study were sourced from the same production batch and managed centrally.

Based on the pharmacological characteristics of the supplements, the following standardized supplementation protocols were implemented:

(1) Acute CAF supplementation protocol: CAF was administered according to body mass at a dose of 3 mg·kg^−1^, ingested once 30 min before each testing session.
(2) Chronic RHO supplementation protocol: RHO was administered as an extract capsule standardized using salidroside as the marker compound and was supplemented continuously throughout the 4-week intervention period. The total daily dose was 2.4 g (corresponding to a labeled total salidroside content of 12 mg·day^−1^), divided into two doses and consumed in the fasted state, 30 min before breakfast and lunch [17].

To maintain consistency in dosing frequency and capsule number across groups during the intervention period, the protocols for each group were as follows:

- RHO + CAF group: continuous RHO supplementation for 4 weeks according to the RHO protocol, with additional acute CAF supplementation on testing days according to the CAF protocol.
- RHO group: continuous RHO supplementation for 4 weeks according to the RHO protocol, with ingestion of placebo capsules matched in appearance to CAF on testing days.
- CAF group: ingestion of placebo capsules matched in appearance to RHO on non-testing days, with acute CAF supplementation on testing days according to the CAF protocol.
- CTR group: ingestion of placebo capsules matched for timing and capsule number throughout both the intervention and testing periods.

Participant compliance was assessed using daily intake logs and capsule return counts (pill count).

### 2.5. Training Program

During the 4-week intervention period, all participants continued to take part in the regular soccer-specific training program routinely organized for the Beijing Sport University varsity team, in order to ensure consistent training exposure and training stimulus across groups. The research team did not impose any additional intervention on the team’s scheduled training content; however, training frequency, session duration, and content structure within each weekly microcycle were recorded and checked for consistency (the training schedule is presented in Table 2) to reduce the potential confounding influence of training variation on the evaluation of the supplementation effects [18].

To minimize the short-term interference of acute training load on testing performance, all testing and data collection sessions were scheduled outside regular training days, and participants were instructed to avoid any high-intensity soccer-specific training or heavy resistance training within 24 h before each testing session. Pre and Post assessments were completed under training schedules and time conditions that were kept as consistent as possible, in order to improve the comparability of pre- to post-intervention measurements.

### 2.6. Daily Dietary and Lifestyle Control

Throughout the experimental period, all participants were instructed to maintain their habitual dietary patterns and daily lifestyle routines and to avoid intentional changes in total energy intake or macronutrient distribution [19]. The use of any additional nutritional supplements, ergogenic aids, or other stimulant substances was prohibited during the study. In addition, all participants were required to strictly restrict the intake of caffeine-containing foods and beverages and to avoid all sources of caffeine within 48 h before each formal testing session [16].

All participants completed daily dietary records, which were used to estimate total energy, carbohydrate, protein, and fat intake. The research team regularly checked the records for completeness and abnormal fluctuations. The results of dietary intake monitoring across the intervention period are presented in Table 3 and indicated overall consistency among groups. One-way ANOVA showed no significant between-group differences in daily energy, carbohydrate, protein, or fat intake during the intervention period (all *p* > 0.05). To reduce the potential confounding effect of differences in non-training physical activity, participants were instructed to engage only in their regular soccer training and the predefined study testing tasks during the experimental period, to avoid any additional high-intensity physical activity, and to maintain a monitored baseline level of daily activity.

To improve consistency in testing conditions, all Pre and Post assessments were conducted between 15:00 and 17:00, and each participant was tested at approximately the same time of day (±1 h) across sessions. Participants were instructed to avoid vigorous exercise and alcohol consumption within 24 h before each testing session and, as far as possible, to replicate both the dinner consumed on the evening before the previous test and the pre-test dietary pattern. On the testing day, participants were required to maintain normal hydration status while avoiding excessive fluid or food intake shortly before testing. These procedures were implemented to minimize the potential influence of differences in diet, recovery status, and daily activity on the study outcomes.

### 2.7. Testing Procedures and Standardization

The baseline assessment (Pre) and post-intervention assessment (Post) followed exactly the same standardized procedures. It should be noted that the “fatigue” examined in the present study did not refer to generalized whole-body fatigue, but rather to a task-specific state of localized accumulated fatigue closely related to soccer aerial dueling actions, induced jointly by repeated high-intensity heading take-off tasks and successive aerial duel tasks. Specifically, the repeated heading contact height test, consisting of eight consecutive trials, was used to simulate the accumulation of neuromuscular load associated with repeated jumping for aerial contests within a short period during match play, whereas the standardized heading duel task was used to further replicate the sport-specific fatigue stress imposed by frequent aerial body contact and heading contests in a more game-representative context. On this basis, the present study aimed to evaluate the effects of different supplementation strategies on aerial duel-related performance under this fatigue model.

To strictly control for the influence of circadian rhythm on neuromuscular performance, each participant completed the Pre and Post assessments within the same time window (±1 h), and all participants were instructed to avoid any additional high-intensity training within 24 h before testing. On each testing day, participants entered the testing venue under identical conditions and completed a standardized dynamic warm-up protocol.

To reduce potential learning effects and verify the measurement stability of all test outcomes, all participants completed one standardized familiarization session before the formal baseline assessment. Test–retest reliability was evaluated by comparing the familiarization session with the formal baseline testing session. Reliability outcomes were expressed using intraclass correlation coefficients (ICC), 95% confidence intervals (CI), and coefficients of variation (CV), and the detailed results are presented in Table 4.

To minimize fatigue carryover effects and order-related bias between tasks, all participants completed the tests in a fixed sequence, and standardized passive recovery intervals were provided between major test components to ensure sufficient phosphocreatine resynthesis. The testing sequence was as follows: (1) countermovement jump (CMJ) and take-off kinetics assessment; (2) repeated heading contact height test; (3) ball exit velocity test; (4) standardized live heading duel task; (5) maximal voluntary isometric contraction (MVIC) testing, including neck flexion, neck extension, left lateral flexion, and right lateral flexion; and (6) session rating of perceived exertion (session-RPE). For clarity, participants completed three valid CMJ trials (60–90 s rest between trials; highest value retained for analysis), eight consecutive repeated heading contact height trials (10 s rest between trials; analyzed across repetitions), three valid ball exit velocity trials (30–60 s rest between trials; highest value retained), ten heading duel bouts (20–30 s rest between bouts; success rate calculated as the percentage of successful bouts), and three valid MVIC trials in each neck direction following one familiarization trial (≥60 s rest between trials; highest value retained). Session-RPE was recorded once immediately after completion of the full testing battery. In addition, a standardized passive recovery interval of 3 min was provided between major test components. The testing venue, equipment layout, and operating personnel were kept highly consistent between Pre and Post assessments. All researchers responsible for data collection, recording, and adjudication of duel outcomes remained strictly blinded throughout the study to minimize subjective bias.

### 2.8. Assessment of Lower-Limb Explosive Performance and Take-Off Kinetics: Countermovement Jump (CMJ) and Early Impulse (Impulse0–200)

**Figure 2:**
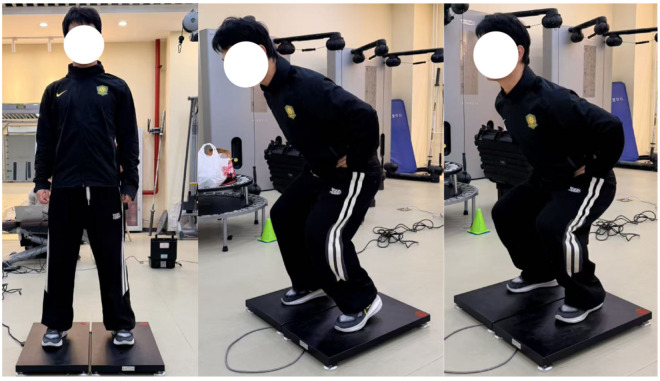
Countermovement Jump and Early Impulse During Take-Off Initiation.

The countermovement jump (CMJ) test was used to quantify lower-limb neuromuscular explosive performance, and force-platform data collected simultaneously were used to derive the early impulse variable during the take-off initiation phase [20]. As shown in Figure 2, testing was performed on a professional three-dimensional force platform (KWFP6035-A2, Shanghai Kunwei Sports Technology Co., Ltd., Shanghai, China), with a sampling frequency of 1000 Hz. Participants were instructed to stand with their feet shoulder-width apart and their hands placed on their hips to eliminate impulse compensation resulting from arm swing. Before the start of each trial, participants remained standing quietly on the force platform for 2–3 s, and the mean vertical ground reaction force vGRF recorded during this quiet standing period was used as the body weight baseline BW. Participants then performed a rapid downward countermovement on command and immediately executed a maximal vertical jump with maximal intended effort. Any form of preparatory hopping or pre-jump movement was strictly prohibited.

For force-platform data processing, the raw vGRF data were used to calculate net force (Net Force):

$$F_{net}=vGRF-BW$$

The onset of take-off (t0) was rigorously defined as the first time point at which vGRF continuously deviated from the BW baseline beyond the predefined threshold. As a key kinetic variable reflecting the initial advantage during take-off initiation, early take-off impulse (Impulse0–200) was defined as the time integral of net force Fnet over the 0–200 ms window following t0 and was normalized to body mass (unit: N·s·kg^−1^) [21].

Peak CMJ height was estimated using the industry gold-standard take-off velocity method [22]. Specifically, instantaneous acceleration was obtained by dividing Fnet by the system mass (m), and vertical velocity of the body center of mass (COM) was then derived by time integration. The maximal vertical take-off velocity (vtake-off) at the instant of take-off, defined as the first sampling point at which vGRF dropped below 20 N, was extracted and entered into the following equation to calculate jump height:

$$h=\frac{\left(v_{take-off}^{2}\right)}{2g}$$

(where g = 9.81 m·s^−2^). Each participant completed three valid jump trials, with a standardized passive rest interval of 60–90 s between attempts. The highest CMJ height obtained across the three trials was retained for statistical analysis [23].

### 2.9. Repeated Heading Contact Height Test

This test was used to evaluate peak heading contact height under a stationary two-foot take-off condition, as well as the maintenance of performance across repeated attempts. Two-dimensional high-speed video acquisition was employed [24]. A high-speed camera (DJI, Shenzhen, China) was mounted on a tripod and positioned approximately 6 m lateral to the participant’s sagittal plane [25]. A vertical jump measuring device with a standardized scale was placed within the same plane of motion to provide two-dimensional spatial calibration. Before testing, a high-contrast marker (diameter: 2 cm) was attached to the center of the participant’s forehead. A standard soccer ball was suspended directly above the take-off area as the contact target, and the suspension height was individualized according to the participant’s standing height and baseline maximal CMJ height.

Participants were required to perform stationary two-foot jumps within a predefined marked area. Natural arm swing was permitted, but horizontal displacement was minimized as much as possible. On the verbal command, participants jumped with maximal effort and were instructed to contact the underside of the target ball using the forehead marker. Each participant completed eight consecutive valid maximal-effort trials, with a passive recovery interval of 10 s between attempts. Trials were considered invalid and repeated if obvious distortions occurred, including premature take-off, foot slippage, or deviation from the designated take-off area [26].

**Figure 3:**
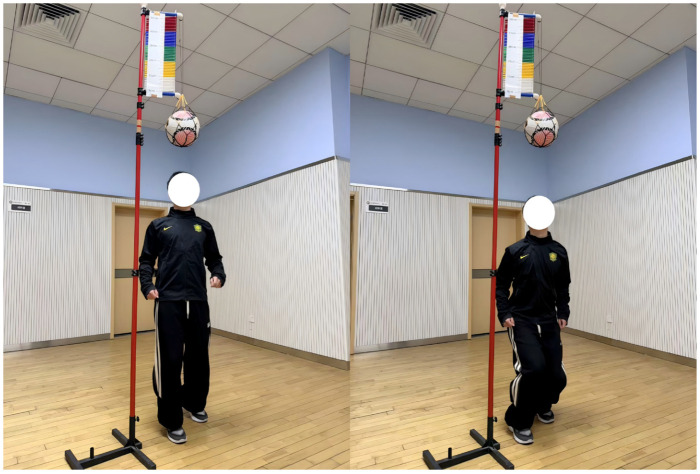
Heading Contact Height Test.

After acquisition, the videos were imported into CapCut (Jianying, ByteDance Ltd., Beijing, China) for frame-by-frame analysis. Using the calibrated reference scale, the highest vertical coordinate (cm) of the forehead marker during each jump was extracted as the peak heading contact height for that trial and was subsequently used for repeated-performance decrement analysis. This eight-repetition sequence was specifically designed to model the cumulative neuromuscular fatigue and task-specific loading patterns typically encountered during frequent aerial contest scenarios in competitive match play, as shown in Figure 3.

### 2.10. Ball Exit Velocity Test

**Figure 4:**
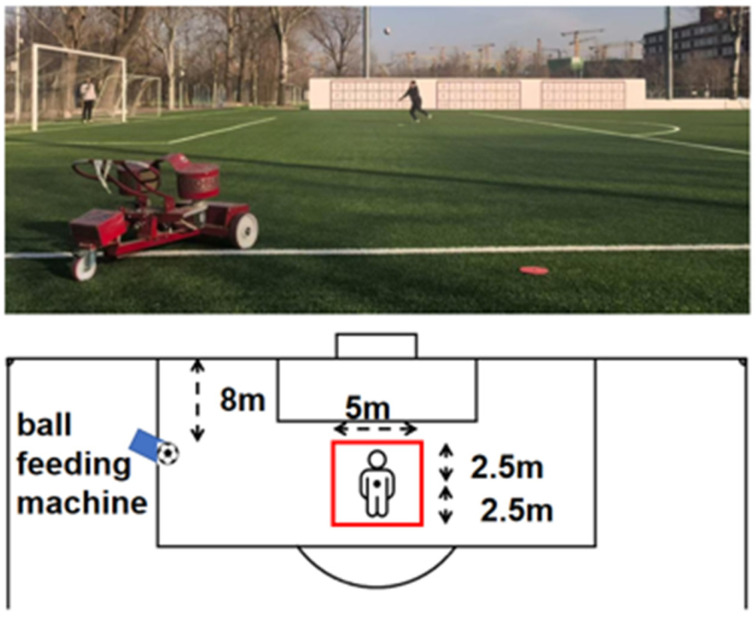
Heading BallExit VelocityTest.

Ball exit velocity during heading was used to assess the efficiency of momentum transfer at ball contact as well as the quality of sport-specific technical execution (Figure 4). Ball delivery was standardized using a soccer ball launcher (EuroGoal 1500, Globus, Codognè, Italy) set at a fixed position to ensure consistency in ball trajectory and landing point [27]. Participants were required to perform the standardized heading action within a designated area (illustrated area width: 5 m, with 2.5 m on each side; approximate distance between the launcher and the participant: 20 m). The same model of standard soccer ball was used throughout testing, and ball pressure was checked before each session to ensure consistency [28].

Ball exit velocity was recorded using a radar gun (10-1911, Bushnell Corporation, Bozeman, MT, USA). The radar device was positioned along the extension of the ball flight path and aligned as closely as possible with the direction of ball travel to minimize angular error. Each participant completed three valid trials; if an obvious execution error occurred or a valid heading action was not achieved, the trial was repeated. A rest interval of 30–60 s was provided between attempts. For statistical analysis, the highest ball exit velocity (m/s) obtained across the three trials was retained as the individual’s maximal sport-specific output. The setup of the ball exit velocity test is shown in Figure 4.

### 2.11. Standardized Heading Duel Success Rate Test

This test was used to quantify the participant’s ability to translate take-off capacity and aerial technical advantage into effective duel outcomes under a standardized one-on-one heading contest condition [29]. To reduce error arising from variation in ball delivery, a ball launcher was used to provide standardized deliveries from a distance of approximately 8 m, with the landing point controlled within a centrally marked area 5 m in width, so as to maintain consistent ball trajectory and landing location across bouts as far as possible [30].

To control contest intensity and reduce the confounding influence of opponent-related differences, each participant was paired with a fixed sparring opponent matched for height, body mass, and years of soccer-specific training, and the same opponent was used for both the Pre and Post assessments. All sparring opponents were blinded to the participants’ supplementation group allocation and were instructed to contest each aerial ball with maximal effort, while prohibited from using illegal physical contact such as pulling or pushing.

Contest success was determined using a binary criterion. A trial was classified as successful if the participant made first contact with the ball before the sparring opponent during the aerial contest and directed the ball into the predefined valid target area (or along the valid trajectory specified by the study protocol). Each participant completed 10 standardized contest bouts, with a passive recovery interval of 20–30 s between bouts. If an obvious anticipatory false start or ball-delivery error occurred, the bout was deemed invalid and repeated. Final heading contest success rate was expressed as the percentage (%) of successful bouts out of the total number of valid bouts.

**Figure 5:**
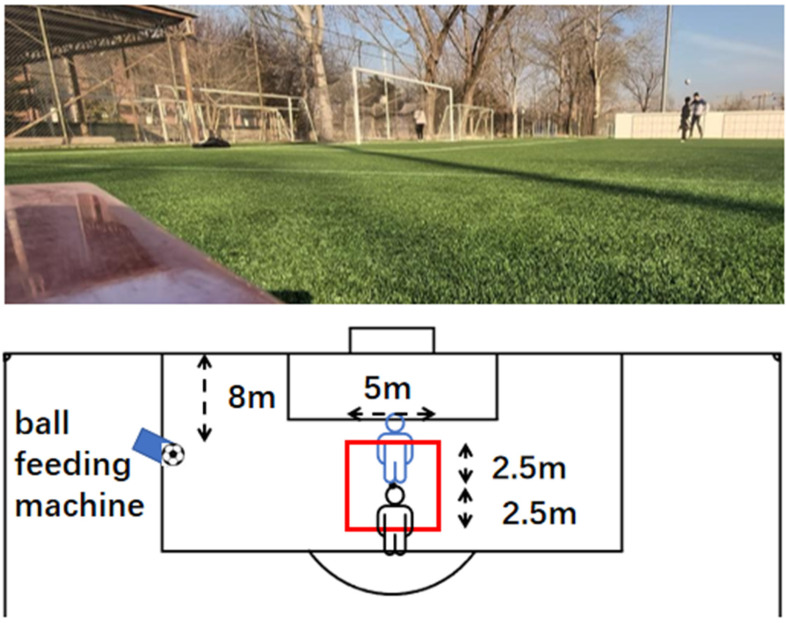
Heading Contest Success Rate Test.

Contest outcomes were recorded independently by two assessors who had undergone consistency training and were blinded to group allocation. In cases of disagreement between the two assessors, the final decision was made by a third reviewer based on high-speed video replay. The setup of the standardized heading duel success rate test is shown in Figure 5.

### 2.12. Assessment of Neck Muscle Strength: Maximal Voluntary Isometric Contraction (Neck MVIC)

Isometric neck strength was assessed to characterize the fundamental neuromuscular capacity underlying stabilization of the head-neck-torso kinetic chain at the instant of ball contact during heading [31]. In the present study, maximal voluntary isometric contraction (MVIC) in neck flexion, neck extension, left lateral flexion, and right lateral flexion was evaluated using a 3D cervical assessment and training system (Multi-Cervical Unit, MCU; BTE Technologies, Hanover, MD, USA). Participants were seated upright in the testing chair, with the thoracolumbar region secured using crossed rigid straps to minimize trunk compensation. Both feet were placed flat on the floor, and both hands firmly grasped the seat handles to stabilize the shoulder girdle. Before testing, system angle sensors were used for calibration to ensure that the head was positioned in the anatomical neutral position (0°). Neck flexion and extension tests were performed in the sagittal plane, whereas left and right lateral flexion tests were performed in the frontal plane [32]. During testing in all directions, compensatory movement in non-target planes, including lateral bending, rotation, or trunk deviation, was continuously monitored and minimized as much as possible.

**Figure 6:**
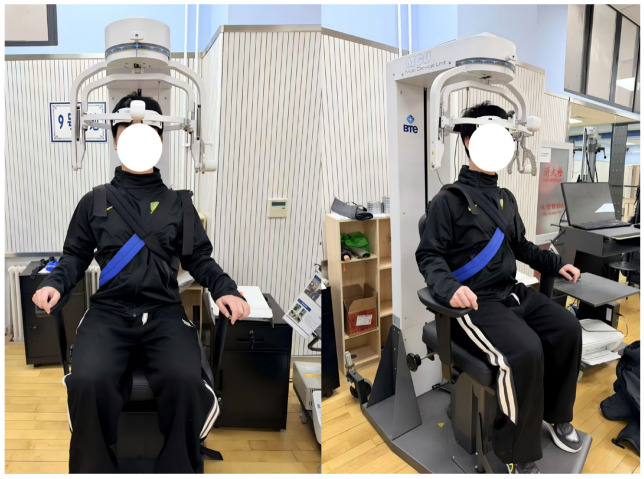
Neck MVIC testing setup.

For each direction, one familiarization trial was performed first and was not included in the analysis. Participants then completed three 5 s maximal-effort MVIC trials, with passive recovery of at least 60 s between trials, while standardized verbal encouragement was provided by the tester. The peak force recorded within each 5 s contraction window was extracted, and the highest value from the three valid trials was retained and normalized to body mass (N·kg^−1^) for statistical analysis. The neck MVIC testing setup is shown in Figure 6.

### 2.13. Assessment of Perceived Internal Load (Session-RPE)

To quantify perceived internal load during the testing process, the present study used a 10-point rating of perceived exertion (RPE) scale (1 = almost no exertion, 10 = extremely strenuous) to record session-RPE [33]. All participants reported one session-RPE value immediately after completing the entire testing battery, which included repeated jumping and heading contest tasks, in order to reflect the overall perceived load of the session. To improve reporting consistency, participants received standardized instructions and anchoring-point explanations for the scale before the baseline assessment, ensuring that they clearly understood the subjective meaning associated with each score.

### 2.14. Statistical Analysis

An a priori sample size calculation was conducted using G*Power software (version 3.1) to justify the number of participants included in the study. Assuming a moderate effect size (Cohen’s f = 0.25), a significance level of α = 0.05, and statistical power (1 − β) = 0.80, the estimated minimum total sample size required for a 4 (group) × 2 (time) mixed-design ANOVA was 76 participants. A total of 96 participants were ultimately included (*n* = 24 per group), indicating that the final sample exceeded the minimum required size. Statistical analyses were performed using SPSS Statistics 27.0, and figures were generated using GraphPad Prism 10. Statistical significance was set at *p* < 0.05. All data are presented as mean ± standard deviation (mean ± SD).

Before parametric analyses were conducted, the assumptions underlying the analyses were examined for all continuous variables. Normality was assessed using the Shapiro–Wilk test, and homogeneity of variance across groups was assessed using Levene’s test. For analyses involving within-subject factors with more than two levels, sphericity was assessed using Mauchly’s test; when the sphericity assumption was violated, degrees of freedom were corrected using the Greenhouse–Geisser adjustment.

The primary pre–post outcome variables were analyzed using two-way mixed-design ANOVA (group × time), with group as the between-subjects factor and time (Pre vs. Post) as the within-subjects factor. This analysis was used to examine the main effects of group and time, as well as the group × time interaction. When a significant interaction was observed, simple-effects analyses were performed, followed by Tukey-adjusted post hoc pairwise comparisons.

For repeated heading contact height across the eight heading attempts, a two-way mixed-design ANOVA (group × repetition) was performed, with group as the between-subjects factor and repetition (H1–H8) as the within-subjects factor. Main effects and group × repetition interactions were examined accordingly. When a significant interaction or main effect was identified, Tukey-adjusted post hoc pairwise comparisons were conducted as appropriate.

Effect sizes were reported as partial eta squared (partial η^2^) for ANOVA results and Hedges’ g for pairwise comparisons. Partial η^2^ values of 0.01, 0.06, and 0.14 were interpreted as small, medium, and large effects, respectively, whereas Hedges’ g values of 0.20, 0.50, and 0.80 were interpreted as small, medium, and large effects, respectively.

## 3. Results

### 3.1. Countermovement Jump Height

**Figure 7:**
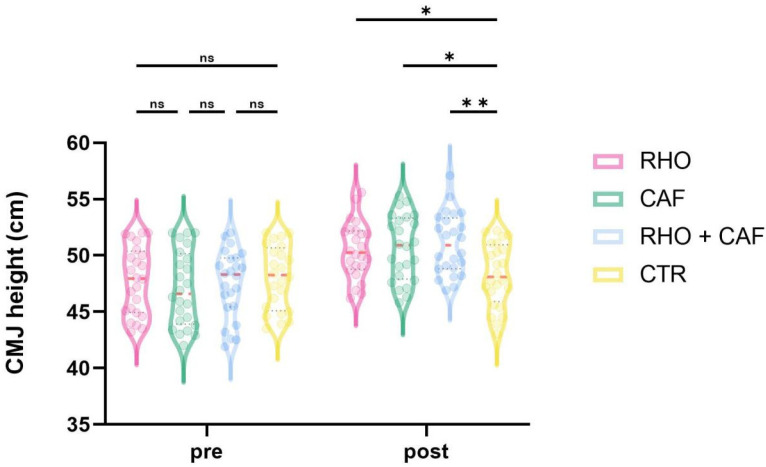
CMJheight (cm). ns, not significant; * *p* < 0.05; ** *p* < 0.01.

As shown in Figure 7, the two-way mixed-design ANOVA revealed a significant group × time interaction effect for CMJ height (*p* = 0.0034, partial η^2^ = 0.138).

Post hoc comparisons at Post showed that the RHO + CAF group had a significantly greater CMJ height than the CTR group (*p* = 0.0065, Hedges’ g = 1.09), and the CAF group also showed a significantly greater CMJ height than the CTR group (*p* = 0.0242, Hedges’ g = 0.95). No other between-group differences at Post reached statistical significance (all *p* > 0.05).

### 3.2. Early Take-Off Impulse (Impulse0–200)

**Figure 8:**
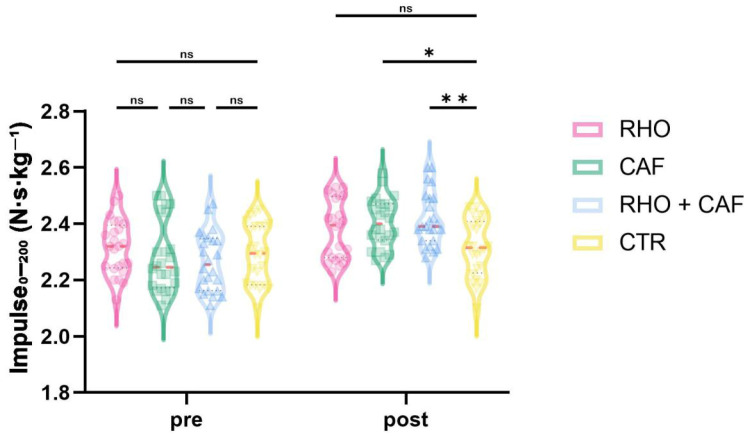
Impulse_0–200_ (N·s·kg^−1^). ns, not significant; * *p* < 0.05; ** *p* < 0.01.

As shown in Figure 8, the two-way mixed-design ANOVA revealed a significant group × time interaction effect for Impulse0–200 (*p* = 0.0007, partial η^2^ = 0.168), indicating that the change in early take-off impulse from Pre to Post differed across supplementation conditions.

Post hoc comparisons at Post showed that the RHO + CAF group had a significantly greater Impulse0–200 than the CTR group (*p* = 0.0037, Hedges’ g = 1.16), and the CAF group also showed a significantly greater Impulse0–200 than the CTR group (*p* = 0.0117, Hedges’ g = 0.88). No other between-group differences at Post reached statistical significance (all *p* > 0.05).

### 3.3. Ball Exit Velocity

**Figure 9:**
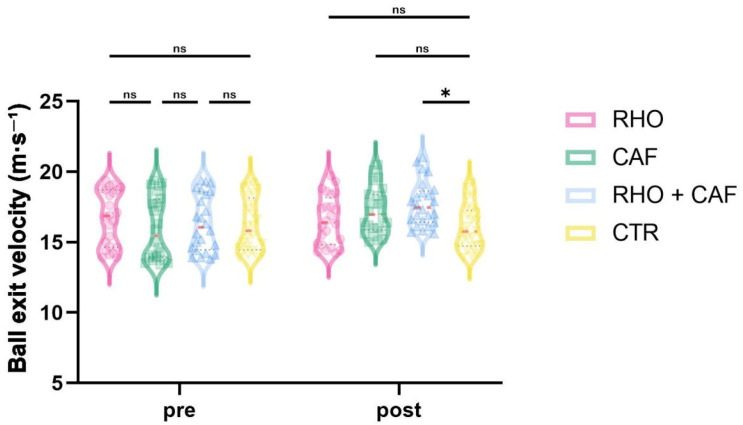
Ball exit velocity (m·s^−1^). ns, not significant; * *p* < 0.05.

As shown in Figure 9, the two-way mixed-design ANOVA revealed a significant group × time interaction effect for ball exit velocity (*p* = 0.0212, partial η^2^ = 0.100).

Post hoc comparisons at Post showed that the RHO + CAF group had a significantly greater ball exit velocity than the CTR group (*p* = 0.0119, Hedges’ g = 1.04). No other between-group differences reached statistical significance (all *p* > 0.05).

### 3.4. Heading Contest Success Rate

**Figure 10:**
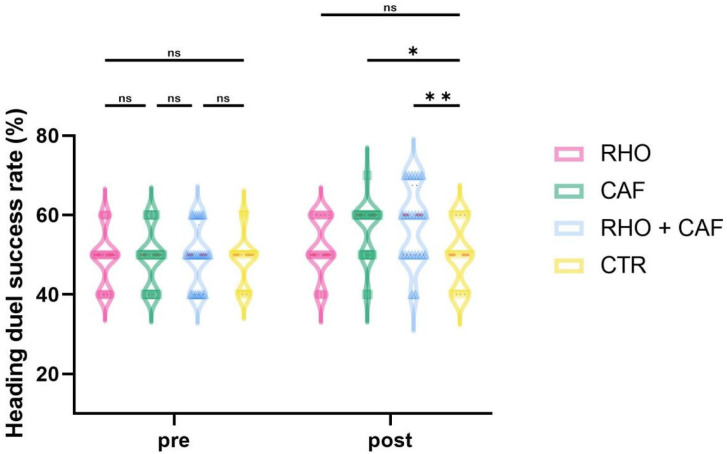
Heading duel success rate (%). ns, not significant; * *p* < 0.05; ** *p* < 0.01.

As shown in Figure 10, the two-way mixed-design ANOVA revealed a significant group × time interaction effect for heading contest success rate (*p* = 0.0145, partial η^2^ = 0.108).

Post hoc comparisons at Post showed that the RHO + CAF group had a significantly greater heading contest success rate than the CTR group (*p* = 0.0036, Hedges’ g = 1.42), and the CAF group also showed a significantly greater heading contest success rate than the CTR group (*p* = 0.0377, Hedges’ g = 1.10). No other between-group differences reached statistical significance (all *p* > 0.05).

### 3.5. Neck Flexion MVIC

**Figure 11:**
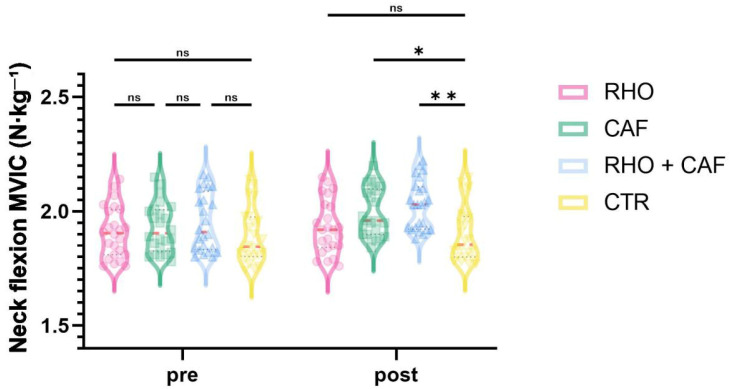
Neck flexion MVIC (N·kg^−1^). ns, not significant; * *p* < 0.05; ** *p* < 0.01.

As shown in Figure 11, the two-way mixed-design ANOVA revealed a significant group × time interaction effect for neck flexion MVIC (*p* = 0.0458, partial η^2^ = 0.083).

Post hoc comparisons at Post showed that the RHO + CAF group had a significantly greater neck flexion MVIC than the CTR group (*p* = 0.0011, Hedges’ g = 1.81), and the CAF group also showed a significantly greater neck flexion MVIC than the CTR group (*p* = 0.0254, Hedges’ g = 1.36). No other between-group differences reached statistical significance (all *p* > 0.05).

### 3.6. Neck Extension MVIC

**Figure 12:**
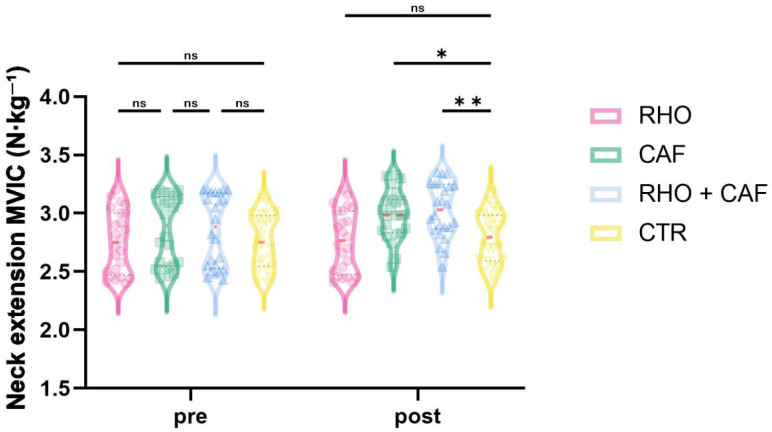
Neck extension MVIC (N·kg^−1^). ns, not significant; * *p* < 0.05; ** *p* < 0.01.

As shown in Figure 12, the two-way mixed-design ANOVA revealed a significant group × time interaction effect for neck extension MVIC (*p* = 0.0168, partial η^2^ = 0.105).

Post hoc comparisons at Post showed that the RHO + CAF group had a significantly greater neck extension MVIC than the CTR group (*p* = 0.0099, Hedges’ g = 1.60), and the CAF group also showed a significantly greater neck extension MVIC than the CTR group (*p* = 0.0402, Hedges’ g = 1.35). No other between-group differences reached statistical significance (all *p* > 0.05).

### 3.7. Left Lateral Flexion MVIC

**Figure 13:**
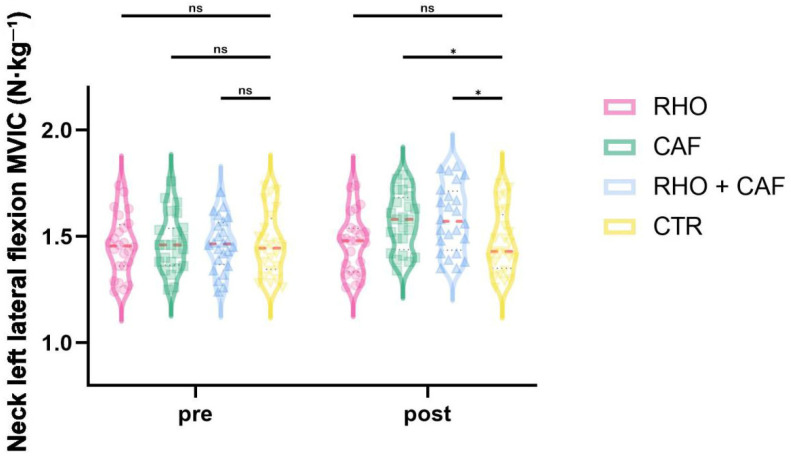
Neck left lateral flexion MVIC (N·kg^−1^). ns, not significant; * *p* < 0.05.

As shown in Figure 13, the two-way mixed-design ANOVA revealed a significant group × time interaction effect for left lateral flexion MVIC (*p* < 0.0001, partial η^2^ = 0.436).

Post hoc comparisons at Post showed that the RHO + CAF group had a significantly greater left lateral flexion MVIC than the CTR group (*p* = 0.0179, Hedges’ g = 0.78), and the CAF group also showed a significantly greater left lateral flexion MVIC than the CTR group (*p* = 0.0494, Hedges’ g = 0.67). No other between-group differences reached statistical significance (all *p* > 0.05).

### 3.8. Right Lateral Flexion MVIC

**Figure 14:**
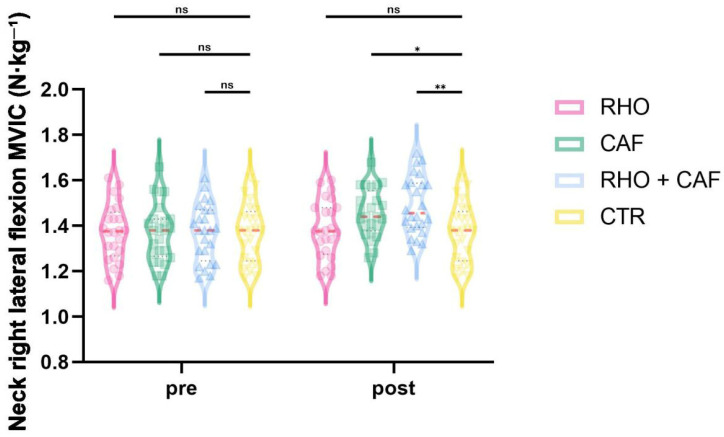
Neck right lateral flexion MVIC (N·kg^−1^). ns, not significant; * *p* < 0.05; ** *p* < 0.01.

As shown in Figure 14, the two-way mixed-design ANOVA revealed a significant group × time interaction effect for right lateral flexion MVIC (*p* < 0.0001, partial η^2^ = 0.273).

Post hoc comparisons at Post showed that the RHO + CAF group had a significantly greater right lateral flexion MVIC than the CTR group (*p* = 0.0025, Hedges’ g = 0.96), and the CAF group also showed a significantly greater right lateral flexion MVIC than the CTR group (*p* = 0.0445, Hedges’ g = 0.69). No other between-group differences reached statistical significance (all *p* > 0.05).

### 3.9. Session-RPE

**Figure 15:**
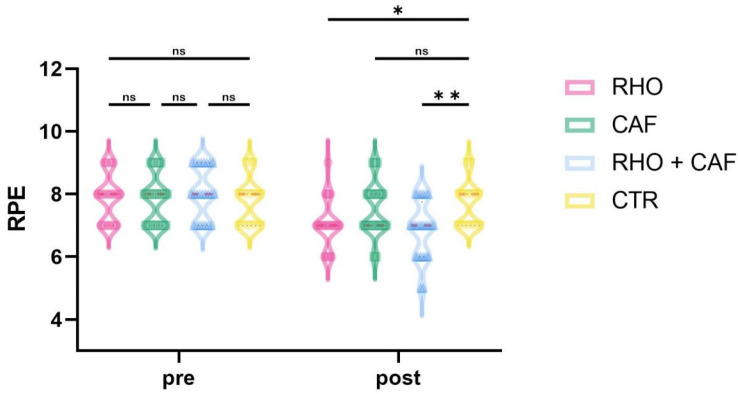
RPE. ns, not significant; * *p* < 0.05; ** *p* < 0.01.

As shown in Figure 15, the two-way mixed-design ANOVA revealed a significant group × time interaction effect for session-RPE (*p* < 0.0001, partial η^2^ = 0.375).

Post hoc comparisons at Post showed that the RHO + CAF group had a significantly lower session-RPE than the CTR group (*p* = 0.0017, Hedges’ g = 2.01), whereas the CAF group showed a significantly lower session-RPE than the RHO + CAF group (*p* = 0.0189, Hedges’ g = 1.61). In addition, the RHO group also exhibited a significantly lower session-RPE than the CTR group (*p* = 0.0318, Hedges’ g = 1.51). No other between-group differences reached statistical significance (all *p* > 0.05).

### 3.10. Pre-Intervention Heading Contact Height

**Figure 16:**
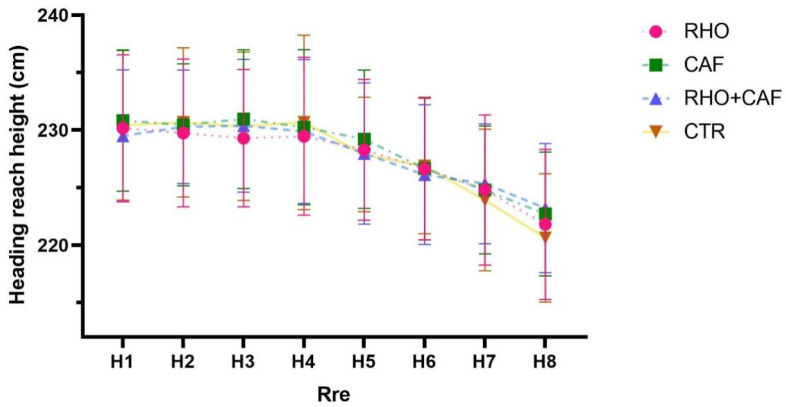
Heading reach height (cm, Pre).

As shown in Figure 16, the two-way mixed-design ANOVA revealed no significant group × repetition interaction effect for heading contact height (*p* = 0.9855, partial η^2^ = 0.014), and the main effect of group was also not statistically significant (*p* = 0.9511, partial η^2^ = 0.004).

However, a significant main effect of repetition was observed (Greenhouse–Geisser corrected: F(4.677, 430.3) = 46.66, *p* < 0.0001, partial η^2^ = 0.337), indicating that heading contact height changed significantly across repeated trials in all groups. Post hoc multiple-comparison analyses showed that no significant between-group differences were observed at any repetition point (H1–H8) (all *p* > 0.05).

### 3.11. Post-Intervention Heading Contact Height

**Figure 17:**
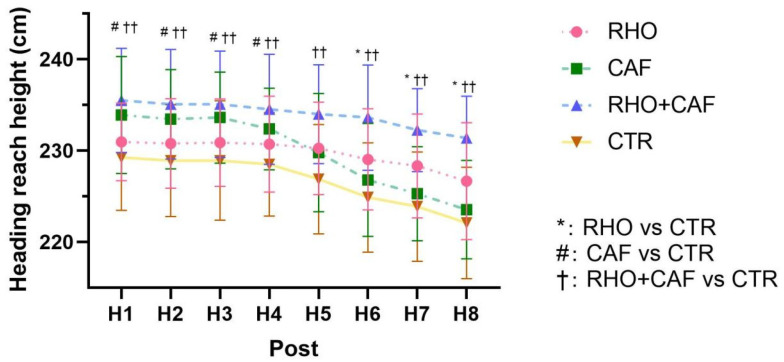
Heading reach height (cm, Post).

As shown in Figure 17, the two-way mixed-design ANOVA revealed a significant group × repetition interaction effect for heading contact height at Post (*p* < 0.0001, partial η^2^ = 0.097). In addition, both the main effect of repetition (*p* < 0.0001, partial η^2^ = 0.338) and the main effect of group (*p* < 0.0001) were statistically significant.

Post hoc comparisons showed that during H1–H4, heading contact height was significantly greater in both the CAF group and the RHO + CAF group than in the CTR group (CAF: *p* = 0.0297, 0.0250, 0.0203, and 0.0310; RHO + CAF: *p* = 0.0014, 0.0028, 0.0034, and 0.0024), whereas no significant differences were observed between the RHO group and the CTR group (all *p* > 0.05). At H5, only the RHO + CAF group showed a significantly greater heading contact height than the CTR group (*p* = 0.0002). During H6–H8, both the RHO group and the RHO + CAF group exhibited significantly greater heading contact height than the CTR group (RHO: *p* = 0.0419, 0.0297, and 0.0388; RHO + CAF: all *p* < 0.0001), whereas no significant differences were observed between the CAF group and the CTR group (all *p* > 0.05).

## 4. Discussion

Under tightly controlled and standardized training exposure over the 4-week intervention period, the present randomized, double-blind, placebo-controlled parallel-group study systematically evaluated the effects of *Rhodiola rosea* (RHO), caffeine (CAF), and their combined supplementation (RHO + CAF) on soccer-specific heading and aerial duel performance. To our knowledge, this is among the earlier studies to systematically compare the effects of CAF, RHO, and their combination within a repeated aerial duel model designed to closely simulate match-relevant fatigue. Overall, the findings were broadly consistent with our initial hypotheses. They suggest that soccer aerial dueling should not be viewed as a single explosive task but rather as a complex performance system involving take-off initiation, repeated-performance maintenance, head-neck stability, and the translation of these capacities into sport-specific outcomes. The effects of the different supplementation strategies showed a differentiated pattern within this system. CAF was primarily associated with improved early kinetic output during movement initiation [34]; RHO was mainly associated with reduced perceived exertion and better maintenance of performance during the later stages of repeated tasks [35]. By contrast, RHO + CAF demonstrated broader and more consistent advantages across multiple variables, including lower-limb explosive performance, multi-directional neck maximal voluntary isometric contraction (MVIC), ball exit velocity, and heading contest success rate. These findings suggest that the benefit of combined supplementation may not reflect a simple additive effect. Instead, it may indicate a potentially complementary pattern between enhanced initiation-related output and improved maintenance-related stability [36].

The significant improvements observed in the CAF group for countermovement jump (CMJ) height and early take-off impulse (Impulse0–200) indicate that its primary benefit was concentrated in the early phase of movement initiation (CMJ: *p* = 0.0034; Impulse0–200: *p* = 0.0007), which is consistent with the central stimulatory effects of caffeine as a classic adenosine receptor antagonist [37]. CAF may attenuate central inhibition, increase neural drive efficiency, and enhance the recruitment of high-threshold motor units, thereby augmenting instantaneous explosive output during take-off initiation and providing an initial temporal and spatial advantage in aerial contests. This mechanism is also consistent with the direction of improvement observed in heading contest success rate in the CAF group (*p* = 0.0145) [38]. Notably, however, the advantage of CAF in the repeated heading contact height test was mainly evident during the early trials and did not persist consistently into the later phase (early repetitions: *p* < 0.05; later repetitions: *p* > 0.05). One possible explanation is that CAF may preferentially enhance neural activation efficiency during the initial phase of movement [39]. However, it may not directly buffer the progressive accumulation of peripheral fatigue during repeated high-intensity tasks. We speculate that, during repeated jumping and contest tasks, the lower-limb extensors and related synergistic muscle groups may gradually experience metabolic by-product accumulation, reduced excitation-contraction coupling efficiency, and impaired contractile function. Under such conditions, central stimulation alone may be insufficient to continuously offset the decline in peripheral mechanical output. Thus, CAF appears to function more as an “initiation-oriented enhancement” strategy than as an intervention that offers equal protection across the entire repeated-performance sequence. This pattern is broadly consistent with previous studies showing that caffeine is more strongly associated with early-phase neuromuscular output and explosive performance than with sustained protection against fatigue-related decline.

In contrast to CAF, RHO displayed a more pronounced “maintenance-oriented” profile. The RHO group did not demonstrate clear advantages in fundamental explosive-performance variables but showed a significant reduction in session-RPE (*p* < 0.0001) and superior performance during the later phase of the repeated heading contact height test (later repetitions: *p* < 0.05), suggesting that its principal role may not be to enhance peak output in a single effort but rather to modulate fatigue perception and preserve performance stability under repeated high-intensity loading [40]. It should be noted that although “adaptogen” is a commonly used term in the literature to describe RHO, from the perspective of exercise physiology, its effects may more plausibly involve modulation of exercise stress responses and central fatigue-related processes. Based on the present findings, one possible interpretation is that RHO may influence stress-response pathways during repeated high-intensity exercise [11,41]. For example, it may be associated with modulation of hypothalamic–pituitary–adrenal (HPA) axis activity or fatigue-related neurotransmitter balance, thereby contributing to reduced perceived exertion and delayed performance decline. Although the present study did not directly assess cortisol, catecholamines, or neurotransmitter-related markers and therefore cannot verify these mechanisms, the consistency between the reduction in session-RPE and the better maintenance of late-stage performance in the RHO group at least suggests that its value in repeated high-intensity sport-specific tasks may lie more in an anti-fatigue maintenance effect than in a traditional acute stimulatory effect. This finding is also in line with previous literature suggesting that RHO may be more closely related to fatigue attenuation, stress-response regulation, and perceived exertion control than to acute enhancement of peak explosive output.

A notable finding of the present study was the broader advantage observed with combined supplementation (RHO + CAF). Compared with either supplement alone, RHO + CAF showed benefits in both the early and later phases of the repeated heading contact height test (all *p* < 0.05) and also produced more consistent improvements in variables more directly related to sport-specific outcomes, including multi-directional neck MVIC (all *p* < 0.05), ball exit velocity (*p* = 0.0212), and heading contest success rate (*p* = 0.0145) (Supplementary Table S1). These performance outcomes directly support the interpretation that combined supplementation was associated with a more comprehensive benefit profile than either supplement alone. At the performance level, this pattern is consistent with a potentially complementary “initiation-maintenance” model, in which CAF may be more closely related to early take-off-related output, whereas RHO may be more closely related to preserving performance during repeated efforts [42]. Compared with previous work that has typically examined CAF or RHO in isolation, the present study extends the literature by evaluating their differentiated and combined associations within a soccer-specific repeated aerial duel model.

However, this interpretation remains inferential because the present study did not directly assess the underlying physiological mechanisms. The increase in multi-directional neck MVIC in the combined group may provide an important clue for understanding how physical output is translated into heading-specific performance [43]. Heading is a complex collision-related action involving body elevation, aerial contest, and ball contact by the head, and its performance depends not only on take-off capacity but also on effective control of the head-neck-torso system at the instant of ball contact [44]. From a biomechanical perspective, greater multi-directional isometric neck strength may be relevant to head-neck stabilization and may contribute to more effective transfer of whole-body momentum into the heading action, although the available evidence remains mixed rather than definitive [45]. Accordingly, the broader advantages of RHO + CAF in ball exit velocity and heading contest success rate may not be attributable solely to faster or higher jumping. They may also reflect a more effective conversion of physical output into sport-specific performance. Nevertheless, explanations involving enhanced neural drive, head-neck stabilization, or force transmission were not directly tested in the present study and should therefore be regarded as plausible but speculative. Future studies incorporating surface electromyography, three-dimensional kinematics, and collision kinetics are needed to verify these mechanisms more directly.

From an applied perspective, the present findings suggest that different supplementation strategies may be associated with different functional patterns in repeated aerial duel-related tasks. CAF may be more relevant when early explosive output is prioritized, whereas RHO may be more relevant when maintaining performance and reducing perceived exertion across repeated efforts are key concerns. Combined RHO + CAF supplementation may be particularly useful in contexts where players are required to repeatedly perform heading and aerial duel actions under fatigue. However, these practical implications should be interpreted within the boundaries of the present study population, namely male collegiate soccer players.

Several limitations of the present study should also be acknowledged, although these limitations simultaneously provide clear directions for future research. First, the sample was restricted to male collegiate soccer players, and therefore caution is needed when generalizing the findings to female athletes, youth populations, or players of higher competitive levels [46]. Second, the present study primarily examined the combined effect of chronic RHO supplementation and acute CAF supplementation within a 4-week intervention framework. This design is relatively close to real-world supplementation practices in competitive settings and therefore has good ecological validity; however, it also increases the complexity of fully disentangling the mechanisms of the two supplements. Future studies may adopt more refined temporal designs to compare the mechanistic differences between chronic and acute supplementation models. Third, the present study did not include biochemical, neurophysiological, or detailed biomechanical measurements, and thus mechanistic interpretation remains largely inferential. Future work could incorporate blood sampling to assess stress- and fatigue-related metabolic markers, use surface electromyography to quantify co-activation of neck musculature during aerial contests, and employ three-dimensional kinematic and kinetic analyses to verify the hypotheses related to rigid head-neck-torso coupling and effective mass transfer. Fourth, although training exposure, dietary monitoring, testing time windows, and pre-test behavioral restrictions were standardized as far as possible, potentially relevant external factors such as sleep quality, psychological stress, and competition-related load were not directly monitored during the intervention period. Accordingly, residual confounding effects cannot be completely excluded and may have contributed to inter-individual variability in the observed responses.

Despite these limitations, the present study clearly reveals the differentiated functional roles of CAF and RHO in soccer aerial dueling, as well as the potential synergistic advantages of their combined use: CAF appears to preferentially improve rapid neural activation and explosive output during the initiation phase. RHO appears to preferentially support fatigue buffering and performance stability during the later phase of repeated tasks; and their combination may more comprehensively address the composite demands of soccer aerial dueling, including rapid take-off, maintenance of performance across repeated efforts, head-neck stabilization at ball contact, and the translation of these capacities into effective sport-specific outcomes [47]. These findings not only provide new practical implications for soccer-specific nutritional interventions, but also establish a theoretical basis for future research focused on neurophysiological mechanisms, biomechanical evidence chains, and optimization of combined supplementation strategies.

## 5. Conclusions

In male collegiate soccer players, CAF, RHO, and RHO + CAF were associated with differentiated effects on soccer-specific repeated aerial duel performance. CAF was more closely related to initiation-related output, RHO to maintenance-related responses under repeated loading, and their combination to a broader overall benefit profile. The novelty of the present study lies in applying this differentiated supplementation framework to a soccer-specific repeated aerial duel model integrating take-off kinetics, repeated heading performance, neck neuromuscular function, and contest outcomes.

## Figures and Tables

**Table 1:** Baseline characteristics of the participants.

Variable	CTR (*n* = 24)	RHO (*n* = 24)	CAF (*n* = 24)	RHO + CAF (*n* = 24)
Age (years)	22.1 ± 3.4	22.8 ± 3.6	22.4 ± 3.2	22.6 ± 3.3
Height (cm)	176.9 ± 6.1	179.8 ± 5.4	177.3 ± 6.7	180.6 ± 6.9
Body mass (kg)	71.4 ± 7.5	74.2 ± 6.8	72.6 ± 7.9	75.1 ± 7.2
Training experience (years)	6.8 ± 2.1	7.6 ± 1.9	6.9 ± 2.3	7.8 ± 2.0

**Table 2:** Weekly soccer training schedule during the 4-week intervention period.

Training Day	Session Duration (min)	Main Training Content	Primary Focus
Day 1	90	Technical training (passing, shooting, heading); low-intensity tactical drills	Maintenance of soccer-specific technical skills
Day 2	90	High-intensity interval running (acceleration, deceleration, change-of-direction, short sprints); small-sided games	Match-related physical demands and neuromuscular stimulation
Day 3	90	Combined technical–tactical training (set-piece situations, heading duels); moderate-intensity contact drills	Aerial duels and game-specific scenarios
Day 4	90	Competitive drills (small- or half-pitch games); cool-down and recovery activities	Match simulation and fatigue management

**Table 3:** Daily dietary intake during the 4-week intervention period.

Variable	CTR (*n* = 24)	RHO (*n* = 24)	CAF (*n* = 24)	RHO + CAF (*n* = 24)
Energy intake (kcal·day^−1^)	2856 ± 248	2894 ± 261	2821 ± 239	2876 ± 254
Carbohydrate (g·day^−1^)	381.5 ± 34.7	387.9 ± 37.6	378.6 ± 33.8	384.8 ± 35.1
Protein (g·day^−1^)	132.4 ± 12.9	134.8 ± 13.6	131.5 ± 12.4	133.7 ± 13.1
Fat (g·day^−1^)	78.6 ± 8.4	80.1 ± 8.8	77.9 ± 8.1	79.4 ± 8.6

**Table 4:** Test–retest reliability of the primary outcome measures derived from the familiarization and formal testing sessions.

Outcome Measure	ICC	95% CI	CV (%)
CMJ height	0.93	0.86–0.97	3.9
Early take-off impulse	0.91	0.82–0.96	4.8
Repeated heading contact height	0.89	0.79–0.95	5.4
Ball exit velocity	0.92	0.84–0.96	4.3
Neck flexion MVIC	0.90	0.80–0.95	5.6
Neck extension MVIC	0.91	0.83–0.96	5.1
Neck left lateral flexion MVIC	0.87	0.75–0.94	6.4
Neck right lateral flexion MVIC	0.88	0.77–0.94	6.1

Abbreviations: CMJ, countermovement jump; MVIC, maximal voluntary isometric contraction; ICC, intraclass correlation coefficient; CI, confidence interval; CV, coefficient of variation.

## Data Availability

Data Availability Statement: The datasets generated and/or analyzed during the current study are not publicly available due to privacy and ethical restrictions but are available from the corresponding author on reasonable request.

## Supplementary Materials

The following supporting information can be downloaded at: https://www.mdpi.com/article/10.3390/nu18091339/s1, Table S1: Mean ± SD values of the principal outcome measures across groups before and after the intervention.

## Abbreviations

The following abbreviations are used in this manuscript:

RHO	*Rhodiola rosea*
CAF	Caffeine
CTR	Placebo Control Group
MVIC	Maximal Voluntary Isometric Contraction
vGRF	Vertical Ground Reaction Force
CMJ	Countermovement Jump
RPE	Rating of Perceived Exertion
