# Supplementary material for: Effects of Combined Caffeine and Rhodiola rosea Supplementation on Repeated Aerial Duel Performance and Neck Neuromuscular Function in Soccer Players
Source: Nutrients. 2026 Apr 23;18(9):1339. doi: 10.3390/nu18091339 (PMC13164687; doi:10.3390/nu18091339)
Supplement: Supplementary file 1 [file nutrients-18-01339-s001.zip › nutrients-4242027-supplementary.pdf]

Supplementary Table S1. Mean ± SD values of the principal outcome measures across groups before and after the intervention.

Outcome measure	CTR Pre	CTR Post	RHO Pre	RHO Post	CAF Pre	CAF Post	RHO+CAF Pre	RHO+CAF Post
CMJ height (cm)	48.04 ± 2.83	48.28 ± 2.81	47.78 ± 3.00	50.47 ± 2.51	47.05 ± 3.34	50.67 ± 2.91	47.48 ± 3.01	51.03 ± 2.73
Ball exit velocity (m·s⁻¹)	16.19 ± 1.86	16.06 ± 1.59	16.70 ± 1.97	16.54 ± 1.75	15.98 ± 2.22	17.20 ± 1.65	16.36 ± 2.00	17.71 ± 1.60
Heading duel success rate (%)	48.33 ± 6.37	50.00 ± 7.80	48.75 ± 6.80	52.08 ± 7.21	49.17 ± 7.17	55.83 ± 7.17	49.58 ± 7.51	57.50 ± 9.44
Session-RPE	7.75 ± 0.74	7.67 ± 0.70	7.88 ± 0.74	7.04 ± 0.75	7.83 ± 0.76	7.50 ± 0.83	8.04 ± 0.81	6.83 ± 0.92

Variable	CTR (n = 24)	RHO (n = 24)	CAF (n = 24)	RHO+CAF (n = 24)
Energy intake (kcal·day⁻¹)	2856 ± 248	2894 ± 261	2821 ± 239	2876 ± 254
Carbohydrate (g·day⁻¹)	381.5 ± 34.7	387.9 ± 37.6	378.6 ± 33.8	384.8 ± 35.1
Protein (g·day⁻¹)	132.4 ± 12.9	134.8 ± 13.6	131.5 ± 12.4	133.7 ± 13.1
Fat (g·day⁻¹)	78.6 ± 8.4	80.1 ± 8.8	77.9 ± 8.1	79.4 ± 8.6
